# Investigating brain aging trajectory deviations in different brain regions of individuals with schizophrenia using multimodal magnetic resonance imaging and brain-age prediction: a multicenter study

**DOI:** 10.1038/s41398-023-02379-5

**Published:** 2023-03-07

**Authors:** Jun-Ding Zhu, Yung-Fu Wu, Shih-Jen Tsai, Ching-Po Lin, Albert C. Yang

**Affiliations:** 1 Institute of Brain Science, National Yang Ming Chiao Tung University, Taipei, Taiwan; 2 Department of Psychiatry, Beitou Branch, Tri-Service General Hospital, National Defense Medical Center, Taipei, Taiwan; 3 Department of Psychiatry, Taipei Veterans General Hospital, Taipei, Taiwan; 4 Institute of Neuroscience, National Yang Ming Chiao Tung University, Taipei, Taiwan; 5 Digital Medicine and Smart Healthcare Research Center, National Yang Ming Chiao Tung University, Taipei, Taiwan; 6 Department of Medical Research, Taipei Veterans General Hospital, Taipei, Taiwan

**Keywords:** Schizophrenia, Predictive markers

## Abstract

Although many studies on brain-age prediction in patients with schizophrenia have been reported recently, none has predicted brain age based on different neuroimaging modalities and different brain regions in these patients. Here, we constructed brain-age prediction models with multimodal MRI and examined the deviations of aging trajectories in different brain regions of participants with schizophrenia recruited from multiple centers. The data of 230 healthy controls (HCs) were used for model training. Next, we investigated the differences in brain age gaps between participants with schizophrenia and HCs from two independent cohorts. A Gaussian process regression algorithm with fivefold cross-validation was used to train 90, 90, and 48 models for gray matter (GM), functional connectivity (FC), and fractional anisotropy (FA) maps in the training dataset, respectively. The brain age gaps in different brain regions for all participants were calculated, and the differences in brain age gaps between the two groups were examined. Our results showed that most GM regions in participants with schizophrenia in both cohorts exhibited accelerated aging, particularly in the frontal lobe, temporal lobe, and insula. The parts of the white matter tracts, including the cerebrum and cerebellum, indicated deviations in aging trajectories in participants with schizophrenia. However, no accelerated brain aging was noted in the FC maps. The accelerated aging in 22 GM regions and 10 white matter tracts in schizophrenia potentially exacerbates with disease progression. In individuals with schizophrenia, different brain regions demonstrate dynamic deviations of brain aging trajectories. Our findings provided more insights into schizophrenia neuropathology.

## Introduction

Schizophrenia is a chronic brain disorder with both positive and negative symptoms and a global prevalence of approximately 0.7% [1]. The etiology of schizophrenia remains unclear thus far, and no effective treatment for completed prevention or alleviation of schizophrenia is available. Therefore, to further obtain new insights into the pathogenesis of schizophrenia, many neuroimaging-based studies have investigated schizophrenia-associated abnormalities in brain structure and function [2–12]. Individuals with schizophrenia have many brain regions with a smaller-than-average volume [2–4]. Similarly, abnormalities including cortical thinning in different brain regions were also observed in these patients [5, 6]. Diffusion tensor imaging (DTI) studies have reported significant decreases in fractional anisotropy (FA) values in specific brain regions, including the genu of the corpus callosum, right forceps minor, left inferior longitudinal fasciculus, left frontal lobe, and left temporal lobe [7–10]. These findings suggest that individuals with schizophrenia have abnormalities in white matter integrity. Moreover, resting-state functional magnetic resonance imaging (fMRI) studies have reported that compared with healthy controls (HCs), individuals with schizophrenia have abnormal functional connectivity (FC) in many brain regions [11, 12]. He and colleagues indicated increased FC in the left insula and bilateral dorsolateral prefrontal cortex of individuals with schizophrenia [11]. Moreover, another study indicated that individuals with schizophrenia have decreased FC within the language network was found in schizophrenia [12]. These findings jointly suggest that individuals with schizophrenia have deteriorated structure and function in various brain regions, which may manifest as deviations in brain aging trajectories in these brain regions.

With the advancement of artificial intelligence, a neuroimaging-based brain-age prediction approach has been applied in studies on neurological and psychiatric disorders, which investigated whether these diseases cause deviations in brain aging trajectory [13–15]. The difference (termed the brain age gap) between chronological and brain ages was calculated. This index is mainly used to examine whether the neurological and psychiatric disorders of participants are associated with accelerated brain aging [13]. Previous studies on brain-age prediction have also demonstrated that individuals with schizophrenia exhibit deviations in brain aging trajectories, as indicated in both T1-weighted magnetic resonance imaging (MRI) [16–24] and DTI [25, 26] findings. The recent studies used multimodal MRI to evaluate the brain age gap in patients with schizophrenia and obtained results consistent with those of studies using a single neuroimaging modality [27–30].

Compared with HCs, individuals with schizophrenia tend to have a brain age that is older than their chronological age. However, this observation has been based on brain-age prediction models constructed using the whole-brain images and data of each participant. Few studies on schizophrenia have focused on constructing brain-age prediction models based on different brain regions. Kaufmann et al. constructed the regional brain age to assess the different spatial brain age gap patterns across several brain disorders. They found that individuals with schizophrenia had the most pronounced acceleration of brain aging based on the model for frontal features [20]. Man et al. found that the brain age gap had negative relationships with brain volume, including subcortical regions and the prefrontal cortex [21]. Neuroimaging studies have indicated that schizophrenia affects different brain regions differently [2–12]. Moreover, structural and functional brain abnormalities potentially cause deviations in brain aging trajectory [31, 32]. Therefore, the development of brain-age prediction models for different brain regions using different neuroimaging modalities can facilitate an examination of how schizophrenia affects different brain regions, thus yielding more comprehensive insights into the neuropathology of schizophrenia.

In this study, we (1) constructed brain-age prediction models for different brain regions that were each based on T1-weighted MRI, resting-state fMRI, or DTI; (2) examined the impacts of schizophrenia on aging trajectory deviations in different brain regions; and finally, (3) investigated relationships between clinicodemographic characteristics (e.g., illness duration, symptom severity, age of onset, history of nicotine use, body mass index, and antipsychotic equivalent dosage) and brain age gaps for brain regions that aged faster than usual.

## Materials and methods

### Participants

To construct the brain-age prediction models, a total of 230 HCs (mean age: 43.05 ± 15.59 years [range: 20–84 years]; sex distribution: 93 men and 137 women; mean Mini-Mental State Examination [MMSE] score: 29.00 ± 0.98; mean duration of education: 15.89 ± 3.67 years) from the discovery cohort “Taiwan Aging and Mental Illness” (TAMI) were recruited in the training dataset. We also obtained neuroimaging data (i.e., T1-weighted MRI, resting-state MRI, and DTI data) of 194 participants with schizophrenia (SCZ dataset) and 100 HCs (HC dataset) from the TAMI cohort to investigate the differences in brain-age gaps between the two groups. The HC dataset was also used to examine the reproducibility of our brain-age prediction models. We incorporated an additional independent cohort (labeled as the BT cohort), including 50 HCs (BT-HC dataset) and 50 individuals with schizophrenia (BT-SCZ dataset) from the Tri-Service General Hospital Beitou Branch, for a final test of the model’s performance and comparison results. The participants with schizophrenia were diagnosed by two psychiatrists according to the Diagnostic and Statistical Manual of Mental Disorders, Fourth Edition, Text Revision (DSM-IV-TR). The participants with mood components (i.e., schizoaffective disorder) and substance use disorders were excluded. The psychiatrists documented the participants’ antipsychotic information from their medical records. Antipsychotic information was available for only 161 participants with schizophrenia in the both cohorts. The exclusion criterion for HCs was receiving a diagnosis of any psychiatric or neurological disease. All participants were asked to complete the MMSE, which was used to evaluate their general cognitive abilities [33]. In addition, participants with schizophrenia received the Positive and Negative Syndrome Scale (PANSS) to assess the severity of symptoms [34]. All participants provided written informed consent, and this study was approved by the Institutional Review Board of Taipei Veterans General Hospital.

### Image acquisition

The MRI data of all participants were obtained on a 3T MRI scanner (Siemens Magnetom Tim Trio, Erlangen, Germany) equipped with a 12-channel head coil at National Yang Ming Chiao Tung University. The scanning protocols were consistent with those in our previous studies [35–37]. Details of T1-weighted MRI, resting-state MRI, and DTI scanning protocols are provided in the supplementary material.

### Image preprocessing

#### Gray matter map construction

**Fig. 1:**
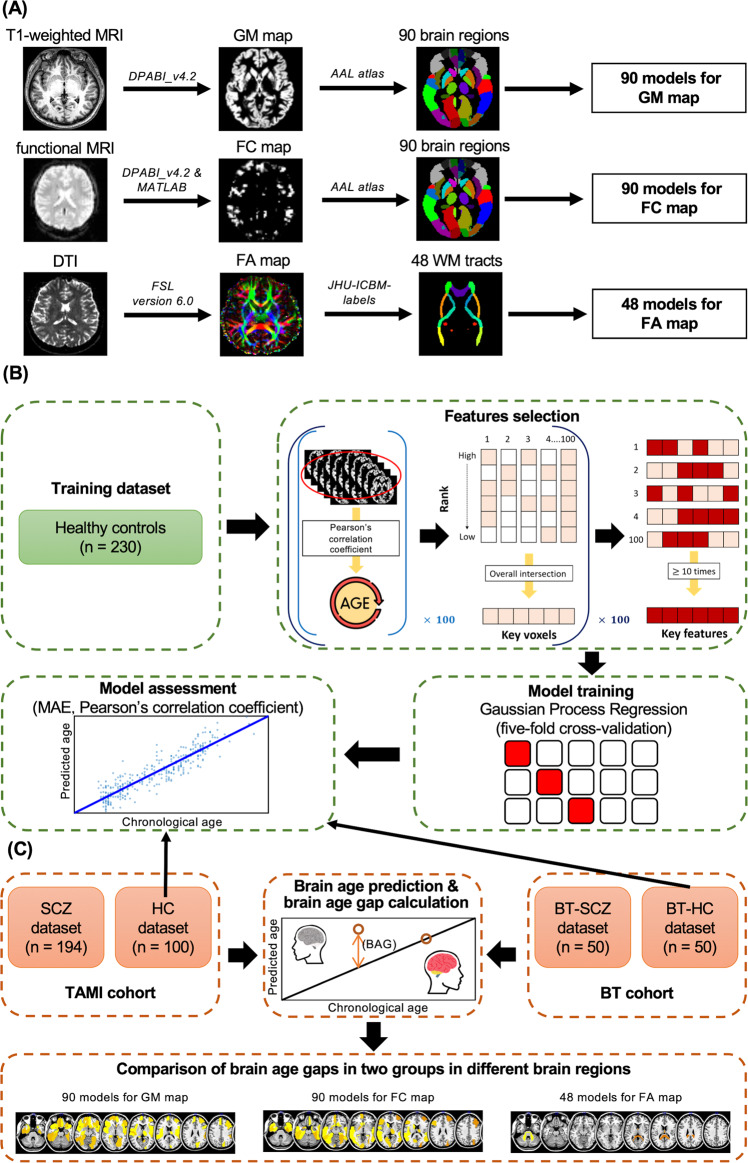
Flow of data processing and brain-age prediction model construction. **A** Illustration of neuroimaging preprocessing steps for T1-weighted MRI, fMRI, and DTI. After data preprocessing, we obtained 90 GM, 90 FC, and 48 FA maps for subsequent analysis. **B** After extracting voxels highly correlated with chronological age for each map as features, we used the Gaussian process regression algorithm to train 228 brain-age prediction models and calculated the MAEs and Pearson’s correlation coefficient between corrected brain ages and chronological ages to evaluate our models’ performance. The HC and BT-HC datasets were also used to examine the reproducibility of our brain-age prediction models. **C** The trained models were applied to the HC, SCZ, BT-HC, and BT-SCZ datasets to estimate brain ages and calculate brain-age gaps. Finally, ANCOVA was used to examine the differences in brain age gaps between participants with schizophrenia and HCs for different brain regions in the TAMI and BT cohorts, respectively. DTI diffusion tensor imaging, GM gray matter, WM white matter, FC functional connectivity, FA fractional anisotropy, AAL automated anatomical labeling, MAE mean absolute error, SCZ individuals with schizophrenia, HCs healthy controls, BAG brain age gap, TAMI Taiwan Aging and Mental Illness, BT Tri-Service General Hospital Beitou Branch.

We used Statistical Parametric Mapping (SPM) 12 and the DPABI toolbox [38] running in MATLAB R2022a (MathWorks, Natick, MA, USA) to preprocess the raw T1-weighted MRI and raw resting-state fMRI data of each participant. For the T1-weighted MRI data, preprocessing proceeded as follows: (1) we reoriented images manually based on the anterior commissure-posterior commissure (AC-PC) line; (2) we normalized all images to the MNI152 standard space and segmented them into gray matter (GM), white matter, and cerebrospinal fluid regions; and (3) we used the automated anatomical labeling (AAL) [39] atlas to further segment the GM images and obtain 90 GM maps for each participant (Fig. 1A).

### FC map construction

We preprocessed the raw resting-state fMRI data per the following steps. First, we removed the first five data points. Second, we corrected all images by slice-timing, realigning, and manually reorienting images. Third, we coregistered the reoriented images with T1-weighted images, normalized to the MNI152 standard space, and resampled them to a 3 × 3 × 3 mm^3^ voxel. Fourth, we regressed out covariates, including those pertaining to the time courses of 6 head motions, white matter, and cerebrospinal fluid. Finally, we also performed temporal lowpass filtering (0.01–0.1 Hz).

Subsequently, we calculated the average voxel-wised FC using the Pearson’s correlation coefficient (*r*) between the blood-oxygen-level-dependent time series of each voxel. We also applied Fisher’s *z* transformation to improve the normality of the data [40, 41]. Finally, we acquired 90 FC maps for each participant by applying the AAL atlas [39] to segment the participants’ images (Fig. 1A).

### FA map construction

We used the FMRIB Software Library v6.0 (FSL) [42] to construct the FA maps. The preprocessing steps were as follows. (1) the eddy currents and movements in the raw DTI data were corrected by the eddy tool [43]; (2) data on the brain tissues were extracted, and data on the nonbrain tissue were removed using the brain extraction tool [44]; (3) FA images were created by fitting the eddy-corrected data into a tensor model at each voxel; (4) the FMRIB58_FA standard-space image was selected as the target to register and align all FA images [45]; and (5) all FA images were normalized to the MNI152 standard space and segmented into 48 FA maps for each participant on the basis of the JHU-ICBM-Labels-1 mm atlas (Fig. 1A).

### Features selection

In order to reduce the irrelevant or partially relevant features, which might negatively impact model performance (e.g., overfitting to the training data) [46, 47], we performed a feature selection procedure for the training dataset of multimodal MRI data in our study. To identify the set of key voxels with the strongest correlations with chronological age in each brain region in all maps (i.e., the 90 GM, 90 FC, and 48 FA maps) for use as the key features, we first randomly selected half of the participants in the training dataset to calculate the Pearson’s correlation coefficient (*r*) between each voxel of different brain regions (i.e., 90 GM and 90 FC maps) and white matter tracts (i.e., 48 FA maps) and chronological age; this step was repeated 100 times. Next, we refined the intersection of 50% of the voxels with the highest *r* value with chronological age in the 100 trials as the key voxels. We then repeated the preceding two steps 100 times to obtain 100 sets of key voxels. Finally, we selected the key voxels that were selected >10 times as the key features of each brain region. Consequently, we established a set of features in 90 GM, 90 FC, and 48 FA maps to construct a predictive model of brain age in different brain regions (Fig. 1B).

### Brain age prediction and brain age gap calculation

The Gaussian process regression algorithm [24, 48–51] with fivefold cross-validation was used to train and assess the 90, 90, and 48 models for GM, FC, and FA maps in the training dataset, respectively. We also applied the trained models to the HC and BT-HC datasets to evaluate reproducibility and predict brain ages in all participants with schizophrenia. To eliminate bias, we corrected all predicted brain ages using the following formulas [52]:

$${\it{Brain}}\,{\it{age = \upalpha \times chronological}}\,{\it{age}} + {\it{\upbeta }}$$

The coefficient α represents the slope and β represents the intercept. This brain age was then corrected as follows:

$${{Corrected}}\,{{brain}}\,{{age}} = {{brain}}\,{{age}}+[{{chronologica}}\, {{age}}-(\alpha \times {{chronological}}\,{{age}}+ \beta)]$$

We calculated the mean absolute error (MAE) and Pearson’s correlation coefficient (*r*) between corrected brain age and chronological age to assess the performance of all models (Fig. 1B). The brain age gaps in different brain regions for the TAMI cohort (194 participants with schizophrenia and 100 HCs) and BT cohort (50 participants with schizophrenia and 50 HCs) were calculated as follows (Fig. 1C):

$${\it{Brain}}\,{\it{age}}\,{\it{gap}} = {\it{corrected}}\,{\it{brain}}\,{\it{age}} - {\it{chronological}}\,{\it{age}}$$

### Statistical analysis

Independent *t* test and Chi-square test were used for the statistical analyses of continuous and categorical clinicodemographic variables, respectively. The *P* value was set at 0.05.

We used analysis of covariance (ANCOVA) to examine the differences in the brain age gaps between participants with schizophrenia and HCs in the TAMI and BT cohorts, with chronological age, sex, MMSE score, and duration of education as the covariates. Moreover, the false discovery rate (FDR) method was used to correct *P* values for multiple comparisons [53]. After FDR correction, the significance level was set at 0.05. The partial eta-squared (partial η^2^) values were calculated as effect size measures. The BrainNet Viewer (http://www.nitrc.org/projects/bnv/) was used for result visualizations [54].

After comparing the brain age gaps between the two groups, we performed a multiple regression analysis for brain regions that aged faster than usual. In each regression model, the dependent variable was brain age gaps for a given brain region; the independent variables were clinicodemographic characteristics, including PANSS subscale scores (for positive symptoms, negative symptoms, and general psychopathology symptoms), illness duration, age of onset, history of nicotine use, and body mass index; and the control variables were chronological age and sex. In addition, after excluding participants without any antipsychotic information and controlling for chronological age and sex, we used a regression analysis to investigate the association between brain age gaps and chlorpromazine (CPZ) equivalent dosage. Finally, the FDR method was used to control for differences in the comparison procedures.

## Results

### Participants’ clinicodemographic characteristics

In the TAMI cohort, the differences between the SCZ dataset (*n* = 194) and the HC dataset (*n* = 100) were nonsignificant in terms of age (*P* = 0.48) and sex (*P* = 0.24). Compared with the HC dataset, the SCZ dataset had worse MMSE scores and shorter durations of education (both *P* < 0.001). Similar to the TAMI cohort, the BT cohort had nonsignificant age (*P* = 0.11) and sex (*P* = 0.69) differences between the two groups. Moreover, the BT-HC dataset had a longer duration of education and MMSE score than did the BT-SCZ dataset (both *P* < 0.001). Table 1 details additional clinicodemographic information in the two datasets.

**Table 1:** Clinicodemographic characteristics of individuals with schizophrenia and HCs in two cohorts.

Characteristics	TAMI cohort				BT cohort			
	SCZ dataset (*n* = 194)	HC dataset (*n* = 100)	Statistic (*t* or χ^2^)	*P*	BT-SCZ dataset (*n* = 50)	BT-HC dataset (*n* = 50)	Statistic (*t* or χ^2^)	*P*
Age, year	43.25 ± 11.93	44.49 ± 15.22	0.71	0.48^a^	47.64 ± 10.42	43.66 ± 13.64	–1.64	0.11^a^
Age of onset, year	27.68 ± 9.35	–	–	–		–	–	–
Sex								
Male, *n* (%)	85 (43.8%)	36 (39.1%)	1.36	0.24^b^	21 (42%)	24 (48%)	0.16	0.69^b^
Female, *n* (%)	109 (56.2%)	64 (60.9%)			29 (58%)	26 (52%)		
Education level, year	12.50 ± 3.55	15.78 ± 4.26	6.98	<0.001^a^	12.08 ± 2.86	14.62 ± 2.48	4.75	<0.001^a^
MMSE	26.81 ± 3.39	28.87 ± 1.10	7.70	<0.001^a^	25.58 ± 4.18	28.94 ± 1.36	5.40	<0.001^a^
Duration of illness, year	15.56 ± 10.30	–	–	–	24.20 ± 8.62	–	–	–
PANSS score								
Total	42.04 ± 10.76	–	–	–	73.16 ± 15.77	–	–	–
Positive symptoms	10.74 ± 3.37	–	–	–	18.14 ± 5.49	–	–	–
Negative symptoms	10.04 ± 3.76	–	–	–	19.42 ± 6.19	–	–	–
General psychopathology symptoms	21.26 ± 5.18	–	–	–	35.60 ± 7.49	–	–	–
CPZ equivalent dosage^c^	402.42 ± 324.27	–	–	–	–	–	–	–
History of nicotine use, *n* (%)	65 (33.5%)	12 (12.0%)	14.27	<0.001^b^	17 (34%)	3 (6%)	10.56	0.001^b^
Body mass index	25.10 ± 4.40	23.35 ± 3.25	3.88	<0.001^a^	25.44 ± 4.92	24.42 ± 3.27	1.22	0.23^a^

*TAMI* Taiwan Aging and Mental Illness, *BT* Tri-Service General Hospital Beitou Branch, *SCZ* individuals with schizophrenia, *HC* healthy controls, *MMSE* Mini-Mental State Examination, *PANSS* Positive and Negative Syndrome Scale, *CPZ* chlorpromazine.

^a^Independent *t* test, significance level = 0.05.

^b^χ^2^ test, significance level = 0.05.

^c^Only 161 participants with schizophrenia had verified medication records in the TAMI cohort.

### Brain-age prediction model performance

In total, 228 brain-age prediction models (i.e., 90 models for GM map, 90 models for FC map, and 48 models for FA map) were trained using the Gaussian process regression algorithm with fivefold cross-validation. In the 90 models for GM map, the results showed consistent MAEs (mean MAE: 6.00 ± 0.38 years, range: 3.90–6.65 years) and strong correlations (mean *r* = 0.90 ± 0.01, range: 0.88–0.95) between the corrected brain and chronological ages (Supplementary Table 1). In the 90 models for FC map, the results revealed that corrected brain ages had strong correlations (mean *r* = 0.97 ± 0.01, range: 0.94–0.99) with chronological ages and low MAEs (mean MAE: 3.28 ± 0.78 years, range: 0.85–4.64 years; Supplementary Table 2). In the 48 models for FA map, we noted consistent MAEs (mean MAE: 5.38 ± 0.70 years, range: 2.55–6.24 years) and strong linear correlations (mean *r* = 0.92 ± 0.02, range: 0.89–0.98) between corrected brain and chronological ages (see Supplementary Table 3 for more details).

We then applied 228 trained models to the HC and BT-HC datasets to verify the reproducibility of the predictions; the results were similar to those of the training dataset (Supplementary Tables 1–3): In the 90 GM, 90 FC, and 48 FA map models, the mean (range) MAEs were, respectively, 6.49 ± 0.64 (4.52–7.86), 3.35 ± 0.81 (1.09–5.52), and 5.63 ± 0.93 (2.98–7.55) years for the HC dataset and 6.15 ± 0.70 (3.78–7.59), 3.91 ± 0.79 (1.89–5.84), and 5.19 ± 0.98 (2.61–7.25) years for the BT-HC dataset; moreover, the mean (range) *r* was, respectively, 0.88 ± 0.02 (0.83–0.94), 0.96 ± 0.02 (0.91–0.99), and 0.92 ± 0.03 (0.86–0.98) for the HC dataset and 0.89 ± 0.03 (0.83–0.95), 0.95 ± 0.02 (0.92–0.99), and 0.91 ± 0.03 (0.83–0.98) for the BT-HC dataset. These results indicated the reliability and consistency of our brain-age prediction models across the different cohorts.

### Comparison of brain age gaps across different brain regions between groups

**Fig. 2:**
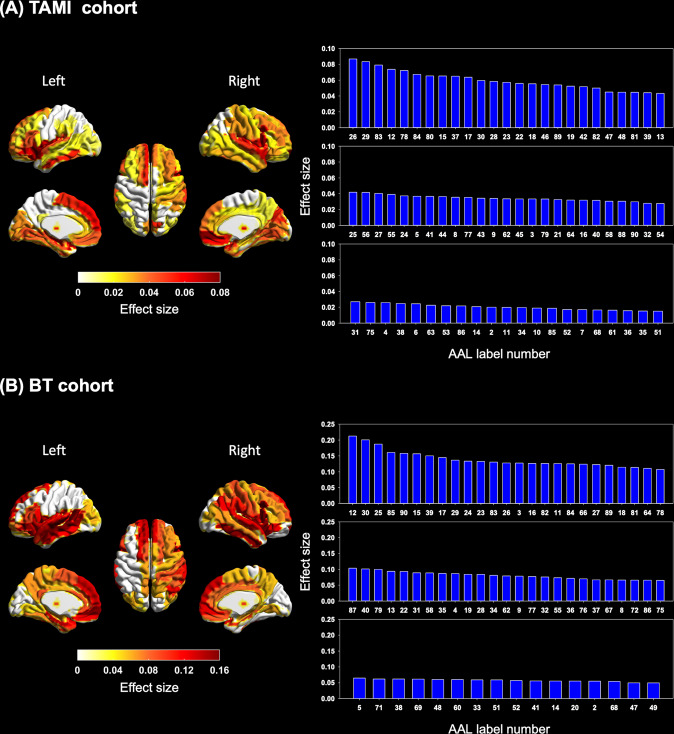
Group differences in brain age gaps between participants with schizophrenia and HCs in 90 models for GM map in the two cohorts. **A** Subplot A illustrates the brain regions with significantly accelerated aging and the effect sizes in participants with schizophrenia in the TAMI cohort. **B** Subplot B presents the brain regions with significantly accelerated aging and effect sizes in participants with schizophrenia in the BT cohort. The brain regions in the left panel display significant differences in brain age gaps after FDR correction in the TAMI and BT cohorts. The color bar represents effect size (partial η^2^). The right panel presents bar charts of effect sizes (partial η^2^ values) for brain regions with significant differences. The brain regions corresponding to the AAL number are presented in Supplementary Table 4. Participants with schizophrenia had significant brain aging trajectory deviations in 71 of the 90 models for GM map in the TAMI cohort. Moreover, 66 brain regions had significantly larger brain age gaps in participants with schizophrenia than in HCs in the BT cohort. We found 10 brain regions with the most pronounced deterioration in both cohorts, occurring primarily in the frontal lobe, temporal lobe, and insula (see Supplementary Table 4). TAMI Taiwan Aging and Mental Illness, BT Tri-Service General Hospital Beitou Branch, FDR false discovery rate, AAL automated anatomical labeling.

In the 90 models for GM map, our results revealed that the participants with schizophrenia had significantly larger brain age gaps than did the HCs in most brain regions after FDR correction in the two cohorts. Of the 90 brain regions, 71 and 66 had significantly larger brain age gaps in the TAMI and BT cohorts, respectively (Fig. 2 and Supplementary Table 4). Of the top 20 brain regions with the largest brain age gap in the TAMI and BT cohorts, 10 of the following brain regions were identified in both the cohorts: the left insula (TAMI cohort: adjusted *P* < 0.001, partial η^2^ = 0.08; BT cohort: adjusted *P* = 0.002, partial η^2^ = 0.14), right insula (TAMI cohort: adjusted *P* < 0.001, partial η^2^ = 0.06; BT cohort: adjusted *P* < 0.001, partial η^2^ = 0.20), opercular part of right inferior frontal gyrus (TAMI cohort: adjusted *P* < 0.001, partial η^2^ = 0.07; BT cohort: adjusted *P* < 0.001, partial η^2^ = 0.21), orbital part of left inferior frontal gyrus (TAMI cohort: adjusted *P* < 0.001, partial η^2^ = 0.07; BT cohort: adjusted *P* = 0.001, partial η^2^ = 0.16), left rolandic operculum (TAMI cohort: adjusted *P* < 0.001, partial η^2^ = 0.06; BT cohort: adjusted *P* = 0.001, partial η^2^ = 0.15), medial part of left superior frontal gyrus (TAMI cohort: adjusted *P* < 0.001, partial η^2^ = 0.06; BT cohort: adjusted *P* = 0.002, partial η^2^ = 0.13), medial orbital part of right superior frontal gyrus (TAMI cohort: adjusted *P* < 0.001, partial η^2^ = 0.09; BT cohort: adjusted *P* = 0.002, partial η^2^ = 0.13), right superior temporal gyrus (TAMI cohort: adjusted *P* = 0.001, partial η^2^ = 0.05; BT cohort: adjusted *P* = 0.002, partial η^2^ = 0.13), temporal poles of left superior temporal gyrus (TAMI cohort: adjusted *P* < 0.001, partial η^2^ = 0.08; BT cohort: adjusted *P* = 0.002, partial η^2^ = 0.13), and temporal poles of right superior temporal gyrus (TAMI cohort: adjusted *P* < 0.001, partial η^2^ = 0.07; BT cohort: adjusted *P* = 0.002, partial η^2^ = 0.13).

The results for 90 models for FC map showed a trend toward greater brain age gaps in the right middle frontal gyrus, the triangular part of the left inferior frontal gyrus, the left supplementary motor area, bilateral parahippocampal gyri, left pallidum, left superior temporal gyrus, and temporal pole of bilateral middle temporal gyri in the participants with schizophrenia compared with HCs in the TAMI cohort. The results for the BT cohort revealed a tendency to increase brain age in the dorsolateral part of the left superior frontal gyrus in patients with schizophrenia relative to the HCs. However, the differences between the two groups in the TAMI and BT cohorts after the FDR correction were nonsignificant (Supplementary Fig. 1 and Supplementary Table 5).

**Fig. 3:**
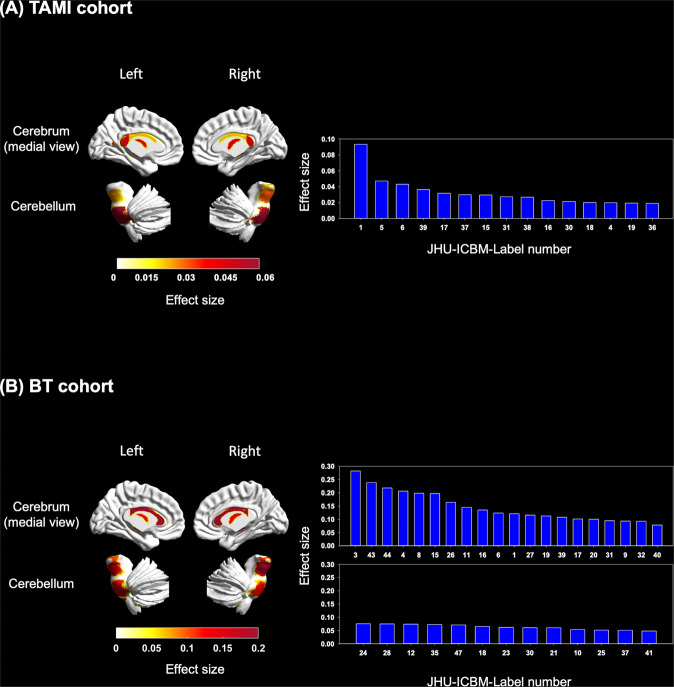
Group differences in brain-age gaps between participants with schizophrenia and HCs in 48 models for FA map in the two cohorts. **A** Subplot A illustrates the white matter tracts with significantly accelerated aging and the effect sizes in participants with schizophrenia in the TAMI cohort. **B** Subplot B presents the white matter tracts with significantly accelerated aging and effect sizes in participants with schizophrenia in the BT cohort. The white matter tracts in the left panel indicate significant differences in brain age gaps after FDR correction in the TAMI and BT cohorts. The color bar represents effect size (partial η^2^). The right panel illustrates bar charts of effect sizes (partial η^2^ values) for white matter tracts with significant differences. The white matter tracts corresponding to the JHU-ICBM-Label number are presented in Supplementary Table 6. Participants with schizophrenia had significantly larger brain age gaps in 15 of the 48 models for FA map in the TAMI cohort. Moreover, 33 white matter tracts had significantly larger brain age gaps in participants with schizophrenia than in HCs in the BT cohort (see Supplementary Table 6). FA fractional anisotropy, TAMI Taiwan Aging and Mental Illness, BT Tri-Service General Hospital Beitou Branch, FDR false discovery rate.

For the TAMI and BT cohorts in the 48 models for FA map, the participants with schizophrenia and HCs significantly differed in the aging trajectory of 15 and 33 white matter tracts, respectively (Fig. 3 and Supplementary Table 6). We identified the largest brain age gap in the TAMI and BT cohorts in the 10 following white matter tracts: the middle cerebellar peduncle (TAMI cohort: adjusted *P* < 0.001, partial η^2^ = 0.09; BT cohort: adjusted *P* = 0.002, partial η^2^ = 0.12), body of corpus callosum (TAMI cohort: adjusted *P* = 0.05, partial η^2^ = 0.02; BT cohort: adjusted *P* < 0.001, partial η^2^ = 0.21), fornix column and body of fornix (TAMI cohort: adjusted *P* = 0.006, partial η^2^ = 0.04; BT cohort: adjusted *P* = 0.002, partial η^2^ = 0.12), left cerebral peduncle (TAMI cohort: adjusted *P* = 0.05, partial η^2^ = 0.02; BT cohort: adjusted *P* = 0.001, partial η^2^ = 0.14), bilateral anterior limb of internal capsule (left: TAMI cohort: adjusted *P* = 0.05, partial η^2^ = 0.02; BT cohort: adjusted *P* = 0.02, partial η^2^ = 0.06. right: TAMI cohort: adjusted *P* = 0.02, partial η^2^ = 0.03; BT cohort: adjusted *P* = 0.02, partial η^2^ = 0.06), left posterior thalamic radiation (TAMI cohort: adjusted *P* = 0.05, partial η^2^ = 0.02; BT cohort: adjusted *P* = 0.03, partial η^2^ = 0.06), right sagittal stratum (TAMI cohort: adjusted *P* = 0.03, partial η^2^ = 0.03; BT cohort: adjusted *P* = 0.006, partial η^2^ = 0.09), right cingulum (hippocampus) (TAMI cohort: adjusted *P* = 0.02, partial η^2^ = 0.03; BT cohort: adjusted *P* = 0.04, partial η^2^ = 0.05), and right fornix cres/stria terminalis (TAMI cohort: adjusted *P* = 0.01, partial η^2^ = 0.04; BT cohort: adjusted *P* = 0.004, partial η^2^ = 0.11).

### Association of deviated brain aging trajectories with clinicodemographic characteristics across different brain regions in participants with schizophrenia

**Fig. 4:**
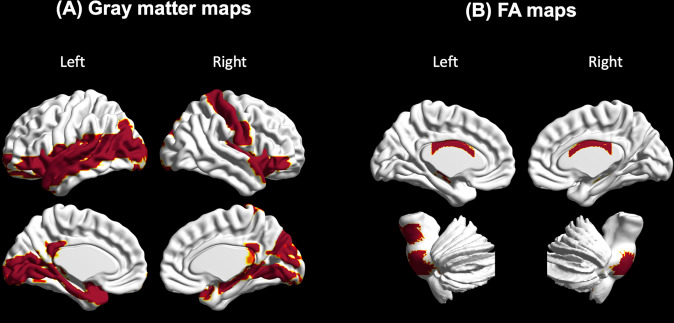
Association of illness duration with brain age gaps across different brain regions in participants with schizophrenia. **A** There were significantly positive correlations between illness duration and brain age gaps in 22 brain regions. **B** Ten white matter tracts were positively associated with illness duration.

We performed multiple regression analysis to further investigate the relationships between brain regions demonstrating accelerated aging and clinicodemographic characteristics of the participants with schizophrenia. The results suggested that illness duration was positively correlated with brain age gaps in 22 GM regions and 10 white matter tracts: bilateral insula (left: beta = 0.23, *T* = 3.16, adjusted *P* = 0.009; right: beta = 0.28, *T* = 3.56, adjusted *P* = 0.004), bilateral posterior cingulate gyri (left: beta = 0.25, *T* = 3.56, adjusted *P* = 0.005; right: beta = 0.21, *T* = 3.20, adjusted *P* = 0.008), temporal poles of bilateral superior temporal gyri (left: beta = 0.31, *T* = 4.11, adjusted *P* = 0.004; right: beta = 0.28, *T* = 3.97, adjusted *P* = 0.003), orbital part of bilateral inferior frontal gyri (left: beta = 0.26, *T* = 3.43, adjusted *P* = 0.006; right: beta = 0.28, *T* = 3.91, adjusted *P* = 0.002), bilateral lingual gyrus (left: beta = 0.28, *T* = 3.74, adjusted *P* = 0.004; right: beta = 0.20, *T* = 2.74, adjusted *P* = 0.024), orbital part of left superior frontal gyrus (beta = 0.19, *T* = 3.07, adjusted *P* = 0.010), left rolandic operculum (beta = 0.30, *T* = 3.39, adjusted *P* = 0.006), right hippocampus (beta = 0.22, *T* = 2.97, adjusted *P* = 0.013), left parahippocampal gyri (beta = 0.25, *T* = 3.27, adjusted *P* = 0.007), left amygdala (beta = 0.30, *T* = 4.11, adjusted *P* = 0.002), left calcarine fissure and surrounding cortex (beta = 0.22, *T* = 3.38, adjusted *P* = 0.006), right cuneus (beta = 0.20, *T* = 3.21, adjusted *P* = 0.008), left middle occipital gyrus (beta = 0.25, *T* = 3.10, adjusted *P* = 0.009), right postcentral gyrus (beta = 0.25, *T* = 3.57, adjusted *P* = 0.005), left superior temporal gyrus (beta = 0.27, *T* = 3.61, adjusted *P* = 0.005), left middle temporal gyrus (beta = 0.25, *T* = 3.11, adjusted *P* = 0.009), temporal pole of left middle temporal gyrus (beta = 0.21, *T* = 3.31, adjusted *P* = 0.007), middle cerebellar peduncle (beta = 0.16, *T* = 2.58, adjusted *P* = 0.020), body of corpus callosum (beta = 0.28, *T* = 4.94, adjusted *P* < 0.001), right inferior cerebellar peduncle (beta = 0.12, *T* = 3.20, adjusted *P* = 0.005), left cerebral peduncle (beta = 0.26, *T* = 3.92, adjusted *P* = 0.001), bilateral posterior corona radiata (left: beta = 0.17, *T* = 3.30, adjusted *P* = 0.007; right: beta = 0.17, *T* = 3.59, adjusted *P* = 0.003), left sagittal stratum (beta = 0.18, *T* = 3.80, adjusted *P* = 0.002), bilateral fornix cres/stria terminalis (left: beta = 0.22, *T* = 4.41, adjusted *P* < 0.001; right: beta = 0.18, *T* = 3.23, adjusted *P* = 0.005), and right tapetum (beta = 0.14, *T* = 3.25, adjusted *P* = 0.006) (Fig. 4).

For the result of age of onset, we found that the age of onset of individuals with schizophrenia had a negative association with the brain age gaps in four white matter tracts, including middle cerebellar peduncle (beta = −0.18, *T* = −3.41, adjusted *P* = 0.01), splenium of corpus callosum (beta = −0.18, *T* = −2.88, adjusted *P* = 0.05), right cerebral peduncle (beta = −0.18, *T* = −2.76, adjusted *P* = 0.05), and left cerebral peduncle (beta = −0.21, *T* = −3.76, adjusted *P* = 0.01).

With regard to symptom severity, nonsignificant associations between PANSS subscale scores and brain age gaps were noted. In addition, our results revealed that CPZ dosages, history of nicotine use, and body mass index had no significant correlations with brain age gaps.

## Discussion

To the best of our knowledge, this is the first study to construct brain-age prediction models based on multimodal MRI data for different brain regions of individuals with schizophrenia and quantify their brain aging trajectory deviations. The data were also derived from cohorts from multiple centers. Individuals with schizophrenia exhibited accelerated aging in brain structures based on GM and FA maps in both the cohorts. Moreover, the brain aging trajectory deviations varied among the brain regions. However, the brain-age prediction models based on FC maps indicated that the brain aging trajectories in the different brain regions of these individuals were similar to those of HCs. In addition, the large brain age gaps in 22 GM regions and 10 white matter tracts in individuals with schizophrenia increased with the progression of the illness.

Our results showed that the individuals with schizophrenia had accelerated aging in most brain regions in models for GM map, with the most considerable deterioration occurring primarily in the frontal lobe, temporal lobe, and insula. Shahab et al. suggested that the most severe brain structural abnormalities related to schizophrenia mainly occurred in the frontotemporal regions [27]. Chen et al. found that cortical thickness significantly contributes to normalized predicted age differences in schizophrenia in the frontal lobe, bilateral precunei, middle temporal gyri, temporal poles, lateral orbitofrontal gyri, and superior parietal gyri [28]. Man et al. found that the subcortical regions and medial and lateral prefrontal cortices had the most significant negative correlations between GM volume and the brain age gap [21]. Kaufmann et al. found that among the different brain regions, the frontal lobe of individuals with schizophrenia had the most pronounced acceleration of brain aging [20]. Zhu et al. revealed that individuals with schizophrenia had significantly larger brain age gaps than those of healthy controls across different durations of illness in the brain volume and cortical thickness models [30]. Neuroimaging-based studies have indicated that GM volume abnormalities mainly occur in the insular cortex, temporal poles, middle cingulum, thalamus, and orbital part of inferior and middle frontal gyri in individuals with schizophrenia [55–57]. The results of a meta-analysis demonstrated that patients with schizophrenia have medium-size volume reductions in bilateral insula, particularly in the anterior insular subregion [58]. These structural imaging-based findings were consistent with our results. These findings jointly suggest that the brain age gap can effectively reflect schizophrenia-related deterioration in brain structure. Moreover, the disease-related structural alteration in the frontal lobe, temporal lobe, and insula may play a critical role in schizophrenia.

Recent studies of brain-age prediction models based on FC maps have focused on participants with preclinical Alzheimer disease and estimated their brain aging trajectory [50, 59]. No study has constructed brain-age prediction models based on FC maps for individuals with schizophrenia. This is the first study to train a machine learning model to predict brain age in individuals with schizophrenia based on FC maps. Although the individuals with schizophrenia exhibited no significantly accelerated brain aging in the 90 models for FC map, we found similar trends in the frontal and temporal lobes; this was similar to the results of our models for GM map. A multimodal MRI study observed acceleration of brain age in young patients with schizophrenia and reported that several brain regions, including the temporal lobe, insula, and parietal lobe, are crucial features in a brain-age prediction model. Different from this study, that study extracted the amplitude of low frequency fluctuation (ALFF), regional homogeneity (ReHo), and degree centrality (DC) values, rather than FC. Therefore, future studies to extract more parameters, including ALFF, ReHo, DC, complexity, and phase coherence to train and predict brain age in schizophrenia, were suggested for providing a more comprehensive understanding of disease-related changes in brain function [29].

Our 48 models for FA map detected 15 white matter tracts that exhibited deviating aging trajectories in the TAMI cohort and 33 white matter tracts that exhibited larger brain age gaps than HCs in the BT cohort. Huang *et al*. extracted the FA values of the fornix column and body of fornix, splenium of corpus callosum, left superior longitudinal fasciculus, and left superior corona radiata as the major features in their prediction model and found that young patients with schizophrenia had aberrant brain aging trajectories [29]. Brain-age studies have indicated that individuals with schizophrenia had a brain age that was older than those of HCs in the FA-based model [25, 26]. Our recent study suggested that there were nonsignificant differences in the global brain age gap between participants with schizophrenia and healthy controls across different illness durations in the FA model [30]. The possible reason is that computing the global brain age gap might reduce the sensitivity for detecting the deviation of aging trajectories of individual white matter tracts. Similar to our findings, several DTI studies have demonstrated that individuals with schizophrenia have lower FA values in the genu and body of corpus callosum, internal capsule, fornix, anterior and superior corona radiata, and cingulum, as found in our findings [60, 61]. The fornix and cingulum (hippocampus), connected to the hippocampus, play essential roles in memory, and patients with neurodegenerative and psychiatric disorders exhibit abnormalities in these regions [62, 63]. In the present study, the middle cerebellar peduncle of the individuals with schizophrenia in both the TAMI and BT cohorts had a relatively older brain age—suggesting that schizophrenia also results in white matter deterioration in the cerebellum, as noted in previous neuroimaging studies [64, 65]. Studies have revealed that the disconnection between the cerebrum and cerebellum might result in FA reduction in the middle cerebellar peduncle along with cognitive impairments in individuals with schizophrenia [64, 65]. Thus, our findings suggested that cerebrocerebellar connectivity disruptions might be involved in the neuropathology of schizophrenia.

Our results indicated that in individuals with schizophrenia, brain aging accelerated with disease progression in 22 brain regions, including the frontal lobe, temporal lobe, parietal lobe, occipital lobe, insula, and subcortical regions; 10 white matter tracts, including the cerebrum and cerebellum, also demonstrated brain aging exacerbation with disease progression. In the TAMI cohort, schizophrenia duration was 15.56 (range, 0–38) years, whereas it was 24.20 (range, 10–45) years in the BT cohort; this might further explain the larger effect sizes in the BT cohort in our GM and FA map models compared with the TAMI cohort. In addition, compared with the TAMI cohort, the BT cohort had more white matter tracts that exhibited aging trajectory deviations. A recent meta-analysis found that compared with those with first-episode schizophrenia, patients with chronic schizophrenia exhibited cortical thinning in the right insula, orbital part of the right inferior frontal gyrus, left lateral middle temporal cortex, and right temporal pole [66]. DTI studies have demonstrated that compared with HCs, individuals with chronic schizophrenia, but not those with first-episode schizophrenia, have lower FA values relative to healthy individuals [8–10]; these results are consistent with ours. In our study, most brain regions exhibited brain aging acceleration in individuals with schizophrenia, but only 22 brain regions and 10 white matter tracts had positive associations with illness duration. In previous studies, brain age gap was nonsignificantly correlated with illness duration because the studies did not construct brain-region-differentiated prediction models of brain age [18, 26, 28].

Our results suggested that individuals with early-onset schizophrenia might have greater deterioration of specific white matter tracts than those of individuals with late-onset schizophrenia. Our findings were similar to a recent brain-age prediction study. Chen *et al*. found that individuals with schizophrenia had a negative relationship between normalized predicted age difference and the age of onset based on the model for white matter [28]. The onset of individuals with schizophrenia often occurs before the full maturation of white matter and is considered a neurodevelopmental disorder [67]. Previous studies found that participants at ultra-high risk of psychosis who later developed psychosis had greater abnormalities of white matter integrity than those who did not transit to psychosis [68, 69]. These findings of an onset-related deterioration in FA indicated that psychosis might result from a stall in white matter maturation [68–70].

The main strength of the current study is the development of brain-age prediction models for different brain regions based on data from T1-weighted MRI, resting-state fMRI, and DTI. Here, we also included two different cohorts to validate and test our models and results. Our methodology could quantify the structural and functional decline of different brain regions in individuals with schizophrenia and provide personalized quantification for clinical explainability. For clinical applications of brain-age prediction in neuropsychiatric disorders, the brain age gap could also serve as an indicator for psychiatrists to assess treatment effects (e.g., whether the brain age gap decreases after the patients receive the treatment). In addition, as opposed to the more abstract concepts of psychiatric disorders and symptoms, the brain-age prediction approach provides patients with a more straightforward understanding of their disease and treatment progression, which may further improve their treatment compliance and insight. Therefore, brain-age prediction is a promising and innovative approach for diagnosing and assessing the course and treatment responses of psychiatric disorders, which could be effectively used in clinical practice. More relevant studies and feasibility studies of brain-age applications in clinical settings are needed in the future.

The limitations of this study are as follows. First, we used a cross-sectional design to construct the brain-age prediction models and examine the brain age gap in our participants with schizophrenia. Nevertheless, future studies should collect longitudinal data to train and validate these brain-age prediction models to more comprehensively understand brain aging trajectories in schizophrenia. Second, our results showed that brain age gaps are nonsignificantly associated with CPZ dosage regardless of brain region. However, antipsychotics might influence brain structure and function in individuals with schizophrenia. In addition, understanding the effects of antipsychotics on brain age is warranted. Third, we only performed the MMSE to assess the overall cognitive function of participants. Previous studies have shown that the increased brain age gap was significantly associated with cognitive decline, including semantic verbal fluency, processing speed, visual attention, cognitive flexibility, spatial Stroop, and symbol coding [71, 72]. However, there were no significant associations between MMSE score and brain gap gaps of all models in both cohorts after performing Pearson’s r correlation analysis and FDR correction (see Supplementary Tables 7–9 for more details). A possible reason is that there was a significant ceiling effect of MMSE, which limited the sensitivity of MMSE in assessing overall cognitive function [73]. Including more cognitive assessments to investigate the relationship between cognitive function and the brain age gap was needed in future studies. Last, since this study was a retrospective study, some clinical factors (e.g., the number of acute episodes/relapses, the number of admissions, socio-occupational functions, and so on) of individuals with schizophrenia could not be further analyzed because these clinical factors were not recorded. Future studies should collect more detailed clinicodemographic data and investigate the associations between these factors and brain age gaps, which might provide more valuable clinical information and applications.

In conclusion, this is the first study to establish brain-age prediction models for different brain regions in individuals with schizophrenia through multimodal MRI. We noted that most GM regions in individuals with schizophrenia exhibit accelerated aging, particularly in the frontal lobe, temporal lobe, and insula and that parts of the white matter tracts, including the cerebrum and cerebellum, demonstrate aging trajectory deviations in individuals with schizophrenia. Notably, the accelerated aging of specific brain regions and white matter tracts worsens with the advancement of illness duration. Moreover, four white matter tracts had negative associations with the age of onset of individuals with schizophrenia. The different brain regions of individuals with schizophrenia differ in their deviations of aging trajectories. By constructing brain-age prediction models for different brain regions, we could quantify the structural and functional deterioration of different brain regions and white matter tracts in schizophrenia. In addition, our methodology could examine the effects of schizophrenia on the dynamics in different brain regions and provide personalized quantification for clinical explainability.

## Supplementary information

SUPPLEMENTAL MATERIAL

## Data Availability

The data presented here are not available due to a confidentiality agreement required by the institutional review board. All MATLAB scripts that were used in the analyses reported in this manuscript are available from the corresponding author upon request.
