# Supplementary material for: Investigating brain aging trajectory deviations in different brain regions of individuals with schizophrenia using multimodal magnetic resonance imaging and brain-age prediction: a multicenter study
Source: Transl Psychiatry. 2023 Mar 7;13:82. doi: 10.1038/s41398-023-02379-5 (PMC9992684; doi:10.1038/s41398-023-02379-5)
Supplement: Supplementary file 1 — SUPPLEMENTAL MATERIAL [file 41398_2023_2379_MOESM1_ESM.docx]

**Investigating brain aging trajectory deviations in different brain regions of individuals with schizophrenia using multimodal magnetic resonance imaging and brain-age prediction: a multicenter study**

– SUPPLEMENTARY MATERIAL –

Jun-Ding Zhu, Yung-Fu Wu, Shi-Jen Tsai, Ching-Po Lin, Albert C. Yang*

**Supplementary methods**

*Image acquisition*

The MRI data of all participants were obtained on a 3T MRI scanner (Siemens Magnetom Tim Trio, Erlangen, Germany) equipped with a 12-channel head coil at National Yang Ming Chiao Tung University. The scanning protocols were consistent with those in our previous studies [1-3]. Details of T1-weighted MRI, resting-state MRI, and DTI scanning protocols are provided in the supplementary material. T1-weighted MR images were obtained using a sagittal 3D magnetization-prepared rapid gradient echo (MPRAGE) sequence, and the parameter settings were as follows: repetition time = 2530 ms, echo time = 3.5 ms, inversion time = 1100 ms, matrix size = 256 × 256, slices = 192, slice thickness = 1 mm, voxel size = 1.0 × 1.0 × 1.0 mm^3^, and flip angle = 7°. For resting-state fMRI images obtained using T2*-weighted gradient-echo-planar imaging (EPI) sequence, the parameter settings were as follows: repetition time = 2500 ms, echo time = 27 ms, matrix size = 64 × 64, voxel size = 3.4 × 3.4 × 3.4 mm^3^, time point = 200, field of view = 200 mm, and flip angle = 77°. Finally, DTI images were acquired with a single-shot spin-echo EPI sequence in the axial plane, and the parameter settings were as follows: repetition time = 11 000 ms, echo time = 104 ms, number of excitations = 3, matrix size = 128 × 128, field of view = 26 cm, slices = 70, slice thickness = 2.0 mm, b-value = 1000 s/mm^2^, thirty isotropic diffusion directions, and three nondiffusion weighted T2 images.

**Supplementary Table 1. Model performances of 90 brain-age prediction models for GM map in training, HC, and BT-HC datasets.**

Abbreviations: GM, gray matter; HC, healthy control; MAE, mean absolute error; r: Pearson's correlation coefficient; TAMI, Taiwan Aging and Mental Illness; BT, Tri-Service General Hospital Beitou Branch.

**Supplementary Table 2. Model performances of 90 brain-age prediction models for FC map in training, HC, and BT-HC datasets**

Abbreviations: FC, functional connectivity; HC, healthy control; MAE, mean absolute error; r: Pearson's correlation coefficient; TAMI, Taiwan Aging and Mental Illness; BT, Tri-Service General Hospital Beitou Branch.

**Supplementary Table 3. Model performances of 48 brain-age prediction models for FA map in training, HC, and BT-HC datasets**

Abbreviations: FA, fractional anisotropy; HC, healthy control; MAE, mean absolute error; r: Pearson's correlation coefficient; TAMI, Taiwan Aging and Mental Illness; BT, Tri-Service General Hospital Beitou Branch.

**Supplementary Table 4. Group differences in brain age gaps between participants with schizophrenia and HCs in 90 models for GM map in the two cohorts.**

Abbreviations: GM, gray matter; SCZ, individuals with schizophrenia; HC, healthy control; SD, standard deviation; TAMI, Taiwan Aging and Mental Illness; BT, Tri-Service General Hospital Beitou Branch; FDR, false discovery rate.

Significant differences after FDR correction are shown in red (adjusted *P* < 0.05).

**Supplementary Table 5. Group differences in brain age gaps between participants with schizophrenia and HCs in 90 models for FC map in the two cohorts.**

Abbreviations: FC, functional connectivity; SCZ, individuals with schizophrenia; HC, healthy control; SD, standard deviation; TAMI, Taiwan Aging and Mental Illness; BT, Tri-Service General Hospital Beitou Branch; FDR, false discovery rate.

Significant differences after FDR correction are shown in red (adjusted *P* < 0.05).

**Supplementary Table 6. Group differences in brain age gaps between participants with schizophrenia and HCs in 48 models for FA map in the two cohorts.**

Abbreviations: FA, fractional anisotropy; SCZ, individuals with schizophrenia; HC, healthy control; SD, standard deviation; TAMI, Taiwan Aging and Mental Illness; BT, Tri-Service General Hospital Beitou Branch; FDR, false discovery rate.

Significant differences after FDR correction are shown in red (adjusted *P* < 0.05).

**Supplementary Table 7. Associations of brain age gaps with MMSE score in participants with schizophrenia in 90 models for GM map.**

Abbreviations: GM, gray matter; MMSE, Mini-mental state examination; SCZ, individuals with schizophrenia; TAMI, Taiwan Aging and Mental Illness; BT, Tri-Service General Hospital Beitou Branch; FDR, false discovery rate.

Significant level after FDR correction is shown in red (adjusted *P* < 0.05).

**Supplementary Table 8. Associations of brain age gaps with MMSE score in participants with schizophrenia in 90 models for FC map.**

Abbreviations: FC, functional connectivity; MMSE, Mini-mental state examination; SCZ, individuals with schizophrenia; TAMI, Taiwan Aging and Mental Illness; BT, Tri-Service General Hospital Beitou Branch; FDR, false discovery rate.

Significant level after FDR correction is shown in red (adjusted *P* < 0.05).

**Supplementary Table 9. Associations of brain age gaps with MMSE score in participants with schizophrenia in 48 models for FA map.**

Abbreviations: FA, fractional anisotropy; MMSE, Mini-mental state examination; SCZ, individuals with schizophrenia; TAMI, Taiwan Aging and Mental Illness; BT, Tri-Service General Hospital Beitou Branch; FDR, false discovery rate.

Significant level after FDR correction is shown in red (adjusted *P* < 0.05)


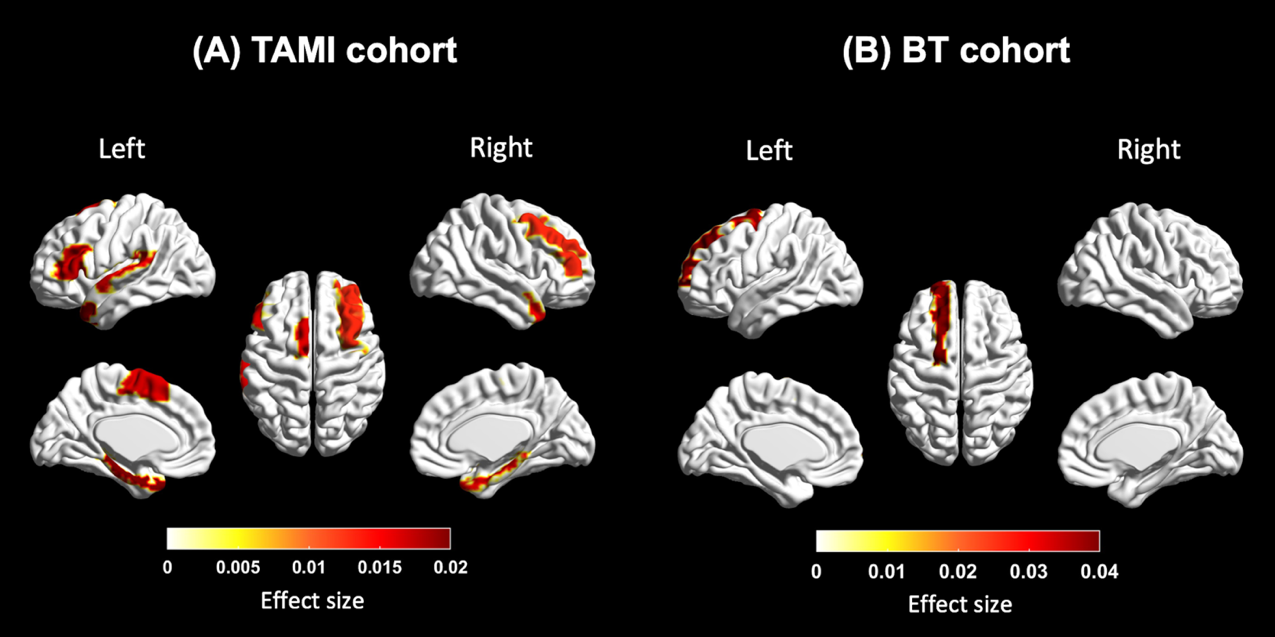


**Supplementary Figure 1. Group differences in brain age gaps between participants with schizophrenia and HCs in 90 models for FC map in the two cohorts.** (A) Subplot A illustrates the brain regions with significantly accelerated aging before FDR correction (uncorrected *P* < .05) and the effect sizes in participants with schizophrenia in the TAMI cohort. (B) Subplot B displays the brain regions with significantly accelerated aging before FDR correction (uncorrected *P* < .05) and effect sizes in participants with schizophrenia in the BT cohort. However, the differences between the two groups after FDR correction were nonsignificant (see Supplementary Table 5). Abbreviations: HCs, healthy controls; FC, functional connectivity; FDR, false discovery rate; TAMI, Taiwan Aging and Mental Illness; BT, Tri-Service General Hospital Beitou Branch.

**References**

1. Yang AC, Huang CC, Yeh HL, Liu ME, Hong CJ, Tu PC, et al. Complexity of spontaneous BOLD activity in default mode network is correlated with cognitive function in normal male elderly: a multiscale entropy analysis. Neurobiol Aging. 2013;34(2):428-38.
2. Yang AC, Huang CC, Liu ME, Liou YJ, Hong CJ, Lo MT, et al. The APOE ɛ4 allele affects complexity and functional connectivity of resting brain activity in healthy adults. Hum Brain Mapp. 2014;35(7):3238-48.
3. Yang AC, Tsai SJ, Liu ME, Huang CC, Lin CP. The association of aging with white matter integrity and functional connectivity hubs. Front Aging Neurosci. 2016;8:143.
